# Baseline malarial and nutritional profile of children under seasonal malaria chemoprevention coverage in the health district of Nanoro, Burkina Faso

**DOI:** 10.1371/journal.pone.0287210

**Published:** 2023-06-26

**Authors:** Paul Sondo, Toussaint Rouamba, Marc Christian Tahita, Karim Derra, Berenger Kabore, Yssimini Nadège Guillène Tibiri, Hyacinthe Abd-El Latif Faïçal Kabore, So-vii Franck Hien, Florence Ouedraogo, Adama Kazienga, Hamidou Ilboudo, Eli Rouamba, Thiery Lefevre, Halidou Tinto

**Affiliations:** 1 Institut de Recherche en Sciences de la Santé (IRSS), Clinical Research Unit of Nanoro (CRUN), Nanoro, Burkina Faso; 2 Institut de Recherche pour le Développement (IRD), Centre National pour la Recherche Scientifique (CNRS), Maladies Infectieuses et Vecteurs: Ecologie, Génétique, Evolution et Contrôle (MIVEGEC), Université de Montpellier, Montpellier, France; Para Federal University, BRAZIL

## Abstract

Seasonal Malaria chemoprevention (SMC) is one of the large-scale life-saving malaria interventions initially recommended for the Sahel subregion, including Burkina Faso and recently extended to other parts of Africa. Initially, SMC was restricted to children 3 to 59 months old, but an extension to older children in some locations was recently recommended. Further characterization of SMC population profile beyond age criterion is necessary for understanding factors that could negatively impact the effectiveness of the intervention and to define complementary measures that could enhance its impact. Children were assessed through a cross-sectional survey during the first month of the 2020 SMC campaign (July-August 2020) as part of the SMC-NUT project in the health district of Nanoro. Parameters such as body temperature, weight, height, mid-upper arm circumference (MUAC) were assessed. In addition, blood sample was collected for malaria diagnosis by rapid diagnostic tests (RDT) and microscopy, and for haemoglobin measurement. A total of 1059 children were enrolled. RDT positivity rate (RPR) was 22.2%, while microscopy positivity rate (MPR) was 10.4%, with parasitaemia levels ranging from 40 to 70480/μL. RPR and MPR increased as patient age increased. Wasting was observed in 7.25% of children under SMC coverage while the prevalence of stunting and underweight was 48.79% and 23.38%, respectively. As the age of the children increased, an improvement in their nutritional status was observed. Finally, undernourished children had higher parasite densities than children with adequate nutritional status. In the health district of Nanoro, children who received Seasonal Malaria Chemoprevention (SMC) were mostly undernourished during the period of SMC delivery, suggesting the need for combining the SMC with synergistic interventions against malnutrition to achieve best impact.

## Introduction

Malaria hitherto affects about 241 million people worldwide, causing 641 000 deaths annually [1]. In Burkina Faso, malaria represents the leading cause of morbidity and mortality [1]. In 2020, up to 3 983 malaria-related deaths were reported in Burkina Faso despite the implementation of multiple control measures, including Seasonal Malaria Chemoprevention (SMC). SMC is one of the most reliable malaria interventions in the Sahel sub-region of Africa [2]. Recently, a flexibility in this geographic restriction was adopted and an extension of this intervention to other parts of Africa was recommended [3]. SMC consist in the administration of a full treatment course of Amodiaquine+Sulfadoxine-Pyrimethamine (AQSP) to children aged 3–59 months on a monthly basis during the high transmission season regardless of malaria infection status [2, 4]. In Burkina Faso, SMC was firstly implemented in 2014 in 6 of the 70 health districts of the country [5, 6]. Few years later, almost national-wide coverage (except in Ouagadougou, i.e. 60 over 70) was reached. However, malaria burden in children remains higher in Burkina Faso suggesting that the expected effect of the SMC intervention in addition to other preventive measures such as the use of bed nets is still not achieved [1]. This implies the existence of hidden factors that could negatively impact on the effectiveness of the intervention. High coverage and good adherence to the intervention were yet reported [7]. Furthermore, AQSP continues to be an effective chemotherapy for malaria with no reported data about a declining efficacy. Investigating other factors that could reduce the expected output of the SMC intervention is crucial and will contribute to determine the complementary measures that could enhance its impact. For instance, there is a strong interplay between malaria and malnutrition; about 50% of malaria deaths are related to malnutrition [8–10]. However, there are controversial findings about the direct relationship between malaria infection and malnutrition. Indeed, while several studies pointed out malnutrition as a risk factor of malaria infection [8, 11–14], other studies reported a protective effect of malnutrition regarding malaria infection [15, 16] or no effect of malnutrition on malaria infection [17, 18]. Beyond all these investigations aimed at establishing the causal relationship, it is crucial to explore the extent to which malnutrition can affect the effectiveness of malaria control interventions such as SMC. This is particularly important because the period of SMC intervention coincides with the period of food shortage in Sahelian countries and SMC target population (children) is also the most affected by malnutrition. In addition, the efficacy of SMC treatment could be jeopardized due to malnutrition. For that reason, mid-upper arm circumference (MUAC) measurement and assessment of edema were recommended during SMC delivery. Furthermore, a previous study highlighted the benefit of packaging SMC with nutrition program in Nigeria [19]. All these aspects justify the necessity of an adequate description of SMC target population in each area. Further characterization of SMC population profile is necessary for understanding host-related factors jeopardizing the impact of the intervention and to smoothly define synergetic measures. Therefore, this study aimed at describing the baseline malarial and nutritional profile of children under SMC coverage in the health district of Nanoro, Burkina Faso.

## Material and methods

### Study area

The study was carried out in four villages of the department of Soaw within the health district of Nanoro (HDN). All the four villages (Soaw, Rakalo, Zoetgomdé and Kakwaka) are located within the health and demographic surveillance system (HDSS) of Nanoro catchment area [20]. The department of Soaw is located at about 25 Km from Nanoro and about 35 Km from Koudougou, the regional health division to which the HDN is affiliated. Two health facilities cover the four villages of the study area: the health facility of Soaw encompassing the village of Soaw, and the village of Rakalo and the health facility of Zoetgomdé encompassing the village of Zoetgomdé and the village of Kalwaka.

Malaria represents a major public health concern with marked seasonal transmission in this area. The high transmission peak occurs during the rainy season, justifying the implementation of the SMC intervention during that period from July to October.

### Study design

This was a community-based intervention as part of the SMC-NUT study, protocol NCT04238845. Details of the SMC-NUT protocol were previously published elsewhere [21]. During the first month of the 2020 SMC campaign, a baseline census was performed by the Nanoro HDSS to identify all eligible children living within the study catchment area. Children whose parent/guardians consented were visited at home by the field workers during the first month of SMC period from July 13^th^ to August 12^th^, 2020. Inclusion criteria were children under SMC and nutrient supplementation coverage (6 months to 5 years old), permanent residence within the study area, ability to complete the study procedures and willingness of parent/ guardian to participate to the study. Children were not included in case of illness at the time of the enrolment, planned travel or inability to complete the study procedure or unwillingness of parents/ guardians to participate to the study. A physical examination was performed to assess parameters such as height, weight, and mid-upper arm circumference measurement (MUAC). Blood sample was taken from finger prick for (i) heamoglobin measurement, (ii) a screening for malaria parasite with RDT and (iii) further microscopical confirmation.

### Assessment of the use of malaria preventive measures

Data on malaria prevention were mainly focused on the use of long-lasting insecticide nets (LLIN). The latter was assessed through the following question addressed to parent/guardian of included children by the field workers: Did the child sleep under an LLIN the night before this visit? A no response was seconded by choice from a drop-down list comprising (i) I have no LLIN in my house, (ii) I have an LLIN in my house, but I have not set it up, (iii) My LLIN is set up, but I have not used it, and (iv) other reason to specify. SMC treatment uptake was not supervised but the number of AQSP tablets taken prior to the time of the field worker’s visit was recorded through self-declaration and examination of remaining doses.

### Screening for malaria infection

Screening for malaria infection was performed by rapid diagnosis tests (RDT) and light microscopy. Histidine rich protein 2 (HRP2) RDTs SD Bioline (SD Standard Diagnostics, INC., Republic of Korea) were used. Malaria slides were stained with 3% Giemsa for 30 mn and double read by two independent microscopists using Olympus Microscope in the parasitology laboratory of the clinical research unit of Nanoro. Body temperature was measured using contactless thermomether Microlife AG, NC 200, Switzerland.

### Assessment of the nutritional status

Photometric measurement of hemoglobin (Hb) in g/dL was performed using an HemoCue® 201+. Hb values were used to define anemia status as follow: No anaemia (Hb≥11.0 g/dL) and anaemia (Hb <11.0 g/dL) [22]. Mid-Upper Arm Circumference (MUAC), considered as the circumference of the left upper arm, was measured at the mid-point between the tip of the shoulder and the tip of the elbow using Shakir trips. Height/lenght was measured using an appropriate Rolla-meter and recorded in centimeters. Body weight was measured using a baby scale for children aged 6 months to 2 years old and a children’s sitting/standing scale for children over 2 years old.

Wasting was defined as a weight-for-height index z scores (WHZ) <-2, while stunting corresponded to a height-for-age index z scores (HAZ)<-2. Underweight was defined as a weight-for-age index z scores (WAZ) <-2 [23].

### Data management and statistical analysis

For the baseline, paper Case Record Form (CRFs) was appropriately developed for each participant and data were double entered in the centralized database using REDCap software. Electronic records were backed up and archived daily. Accuracy, reliability, and consistency of electronic records were verified following a standardized procedures of the CRUN data management team. Any action such as creation, modification, or deletion of electronic records by the operator was independently recorded through a control system. Consistency and completeness of records were judiciously verified before individual records were uploaded and centralized via internet into a central database located in the data management section of the IRSS/CRUN. Furthermore, regular consistency checks were performed by the data manager and all the generated queries were transferred to field supervisors for resolution.

Data analysis was performed using R(version 4.0.5) [24]. We first, performed gender-based comparison of some anthropometric and clinical parameters using student t or Wilcoxon Mann-Whitney tests. Sub-groups were defined regarding malaria infection status (positive *versus* negative) and malnutrition status according to WHZ, HAZ, WAZ and Hb level (Anemia v*ersus* no anemia). Logistic regression by Generalized Linear Models (GLMs) with binomial errors, logit link were used to test the effect of sex, age, villages and interactions on (i) LLIN use, (ii) RDT positivity rate, and (iii) microscopy positivity rate. Binomial GLMs were also used to test the effect of the number of SPAQ tablets ingested on RDT and microscopy positivity rate. Separate binomial GLMs were performed to evaluate the effects of (i) sex, age and villages, and (ii) of number of SPAQ tablets ingested on gametocyte carriage. A negative binomial GLM was used to test the effect of sex, age and villages on parasite density. A negative binomial GLM was also used to test the effect of the number of SPAQ tablets ingested on parasite density. Multiple pairwise comparisons were performed using emmeans [25]. Furthermore, univariate logistic regression was performed to assess the effect of the gender, the age at baseline, the haemoglogin level and nutritional status (stunting, wasting, and underweight) on the malaria infection status and variables with a P value ≤ 0.2 were included in multivariable analyses. The effects of patient age and infection status (as measured by RDT) on (i) stunting, (ii) wasting, (iii) underweight and (iv) malnourished rates were analysed using binomial GLMs. The relationships between parasite density, malnutrition status (stunting, wasting, underweight and malnourished) and age were analysed using negative binomial GLMs. Finally, the effects of patient age and nutritional status (stunting, wasting, underweight and undernourished) on haemoglobin levels were analysed using Gaussian GLMs. For each GLM, the statistical significance of the explanatory variables was evaluated using the “Anova” function of the “car” package.

### Ethical consideration

The study was fully compliant with ICH-GCP ethical principles and national regulations. An informed consent was obtained from the parents/guardians of included children prior to enrolment. The study protocol was registered in clinical Trial.gov registry (NCT04238845) and approved by the ethical committee for health research in Burkina Faso under the reference number N°2020-01-007.

## Results

A total of 1059 children were included in the study. Children’s age was ranged from 0.6 to 5.2 years and all received AQSP during the first round of the 20220 SMC campaign. Gender was equally balanced in the study population, with a sex ratio (M/F) of 1.002 (530 M and 529 F). Table 1 summarizes the socio-anthropometric and clinical parameters of children under SMC coverage in the study area (Table 1).

**Table 1:** Socio-anthropometric and clinical parameters of children under SMC coverage in the health district of Nanoro.

Variable	Overall	Gender		
		Male	Female	p-value
**Age (month)**	** **	** **	** **	**0.13**
n	1059	530	529	
Median (Q25 Q75)	34.80 (24–45)	34.80 (24–43.20)	36 (24–46.80)	
**Weight (kg)**	** **	** **	** **	**0.32**
n	1059	530	529	
Mean (SD)	12.03 (2.73)	12.10 (2.68)	11.95 (2.77)	
**Height (cm)**	** **	** **	** **	**0.08**
n	1059	530	529	
Median (Q25 –Q75)	88 (80–94)	86 (79–93)	88 (80–94)	
**MUAC (cm)**	** **	** **	** **	**0.90**
n	1059	530	529	
Mean (SD)	15.08 (1.27)	15.07 (1.30)	15.08 (1.25)	
**Temperature (°C)**	** **	** **	** **	**0.16**
n	1055	52830	527	
Mean (SD)	35.88 (0.58)	35.91 (0.56)	35.85 (0.59)	
**Haemoglobin (g/dL)**	** **	** **	** **	**0.036**
n	1057	528	529	
Mean (SD)	10.03 (1.42)	9.93 (1.46)	10.12 (1.38)	

Common malaria preventive measure was the use of LLIN. LLIN was highly used and up to 91.0% (962/1057) of children slept under bed nets the night preceding the day of the survey. The majority of non-compliant population had yet the LLIN available in their house. The median number of people sleeping under the same LLIN was 3 (range 1–5). While there was no effect of age or sex, LLIN use varied among villages (89.2, 85.1, 94.3, and 87.3% in Kalwaka, Rakalo, Soaw, and Zoetgomde, respectively, X^2^_3_ = 18.7, p = 0.0003). Hb level was higher in female children than in male children.

**Fig 1:**
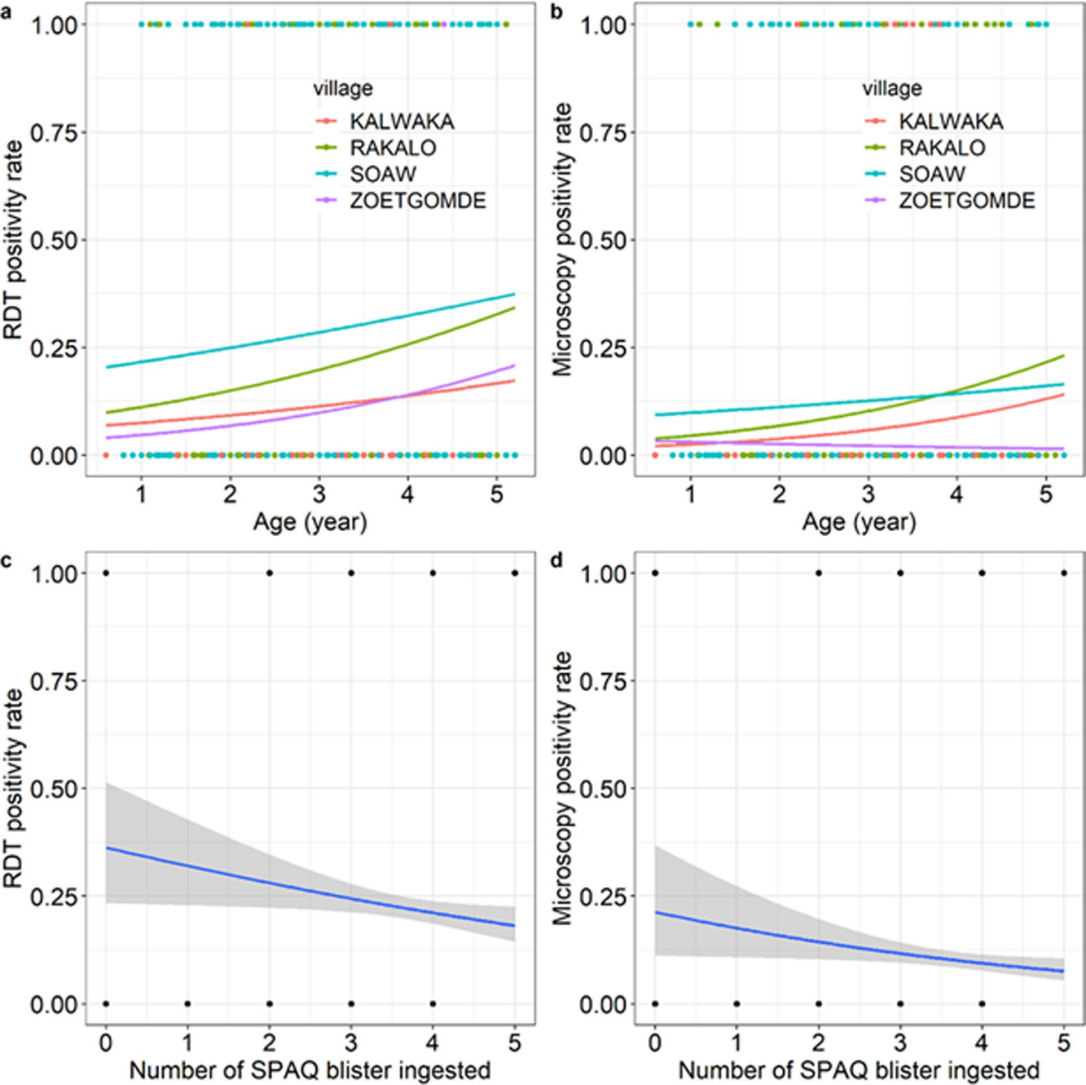
Effects of patient age, village, and number of AQSP tablets ingested on RDT and microscopy positivity rates. (a) RDT positivity rate as a function of patient age for each of the four villages. Each point represents either a positive (1, n = 234) or a negative (0, n = 818) RDT. (b) Microscopy positivity rate as a function of patient age for each of the four villages. Each point represents either a positive (1, n = 110) or a negative (0, n = 949) slide. (c) RDT positivity rate (± 95% confidence interval) as a function of the number of AQSP blister taken. (b) Microscopy positivity rate (± 95% confidence interval) as a function of the number AQSP blister taken.

In the first month of the 2020 SMC campaign, RDT positivity rate (RPR) in children under SMC coverage was estimated at 22.2% (234/1052) and microscopy positivity rate (MPR) was estimated at 10.3% (109/1059). RPR and MPR increased as patient age increased (*X*^*2*^_1_ = 10.5, p = 0.001, Fig 1A, and *X*^*2*^_1_ = 6.15, p = 0.01, Fig 1B). There was also an effect of village on RPR and MPR (*X*^*2*^_3_ = 36.5, p<0.001, Fig 1A, and *X*^*2*^_1_ = 14.7, p = 0.002, Fig 1B), with higher malaria prevalence observed in Soaw and Rakalo compared to Kalwaka and Zoetgomde. There was no effect of sex and no interactions neither on RPR nor on MPR. The number of AQSP tablets ingested ranged from 0 (if blood smears was taken just before the administration of the first dose of AQSP) to 5 (resulting from a re-administration following a rejection). The more AQSP tablets ingested, the less likely it was to be positive by RDT (*X*^*2*^_1_ = 4.6, p = 0.03, Fig 1C) or microscopy (*X*^*2*^_1_ = 4.5, p = 0.03, Fig 1D) (Fig 1).

Parasite density, as measured by microscopy, ranged from 40 to 70480 parasites/μL of blood (median = 1387). There was no effect of patient age on parasite density (*X*^*2*^_1_ = 1.36, p = 0.24). Parasite density was significantly higher in Soaw and Zoetgomde as compared to Kalwaka and Rakalo (*X*^*2*^_3_ = 33, p<0.001), and females harbored higher parasite densities than males (4328.7 ± 852.8 vs 3853.1 ± 1408.2, *X*^*2*^_1_ = 7.63, p = 0.006). There was no effect of the number of ingested AQSP blister on parasite density (*X*^*2*^_1_ = 0.32, p = 0.57). The most frequent parasite species (as measured by microscopy) was by far *Plasmodium falciparum* with 100% (109/109) of infections, followed by *Plasmodium malariae* (4 co-infections with *P*. *falciparum*).

Gametocyte carriage was estimated at 2.36% (25/1059) in children under SMC coverage. Of these 25 gametocyte-positive samples, 11 also harboured asexual stages. There was no effect of village (*X*^*2*^_3_ = 5.15, p = 0.16), age (*X*^*2*^_1_ = 0.25, p = 0.61), sex (*X*^*2*^_1_ = 0.05, p = 0.81) or number of AQSP blister ingested (*X*^*2*^_1_ = 0.54, p = 0.46) on gametocyte carriage.

All cases of malnutrition observed were undernutrition with no case overweight or obesity. Wasting was observed in 7.25% of children under SMC coverage while the prevalence of stunting and underweight was 48.79% and 23.38%, respectively. Undernutrition in all forms was observed in 56.94% of children *versus* 43.06% with adequate nutritional status (ANS).

Table 2 shows the results of the association between undernutrition and malaria infection status using binomial generalized linear model.

**Table 2:** Results of the association between undernutrition and malaria infection status using binomial generalized linear model.

Parameter	Crude OR	95%CI	p-value	Adjusted OR	95%CI	p-value
**Hb level**						
< 11gg/dL	2.50	1.42–4.40	0.001	2.83	1.60–5.01	<0.0001
≥11 g/dL	1					
**Wasting**						
≥-2 z score	1					
<-2 z score	0.64	0.27–1.52	0.322			
**Stunting**						
≥-2 z score	1					
<-2 z score	1.22	0.82–1.81	0.324			
**Underweight**						
≥-2 z score	1			1		
<-2 z score	0.73	0.44–1.21	0.236	0.71	0.43–1.19	0.200

Considering the 3 forms of undernutrition, no direct association with malaria infection was observed. Haemoglobin level represented independent risk factors for malaria infection. No difference between undernourished children and children with ANS was observed on gametocyte carriage (*X*^*2*^_1_ = 1.27, p = 0.25).

**Fig 2:**
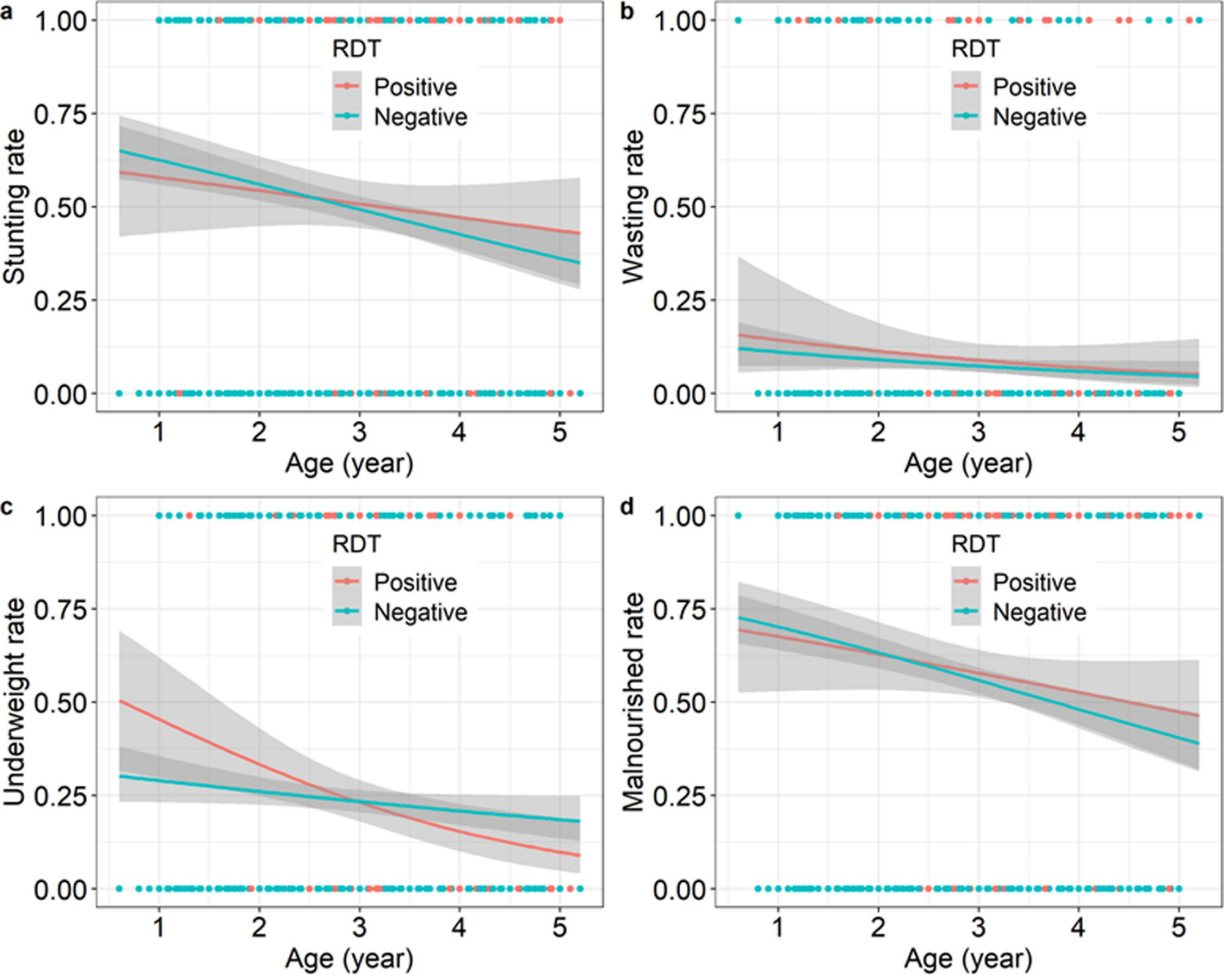
Effects of patient age and infection status (as measured by RDT) on stunting, wasting, underweight and malnourished rates. (a) Stunting rate as a function of patient age and RDT. Each point represents either a positive (1, n = 534) or a negative (0, n = 525) stunting status. (b) Wasting rate as a function of patient age and RDT. Each point represents either a positive (1, n = 85) or a negative (0, n = 974) wasting status. (c) Underweight rate as a function of patient age and RDT. Each point represents either a positive (1, n = 252) or a negative (0, n = 807) underweight status. (d) Malnourished (i.e. stunting or wasting or underweight) rate as a function of patient age and RDT. Each point represents either a positive (1, n = 603) or a negative (0, n = 456) malnourished status.

All three forms of undernutrition (i.e. stunting, wasting, and underweight rates) decreased with patient age (Fig 2, stunting: *X*^*2*^_1_ = 18, p<0.001, wasting: *X*^*2*^_1_ = 4.8, p = 0.028, underweight: *X*^*2*^_1_ = 10, p = 0.002), indicating that as the age of the children increased, there was an improvement in their nutritional status. Although infection status (as measured by RDT) did not influenced stunting and wasting rate, infected individuals were marginally more likely to be undernourished (*X*^*2*^_1_ = 3.9, p = 0.049, Fig 2C). Finally, there also was a marginally significant interaction between patient age and infection status on the underweight rate (*X*^*2*^_1_ = 4.08 p = 0.04, Fig 2C), suggesting that underweight was deteriorated by malaria infection in younger children only (Fig 2).

**Fig 3:**
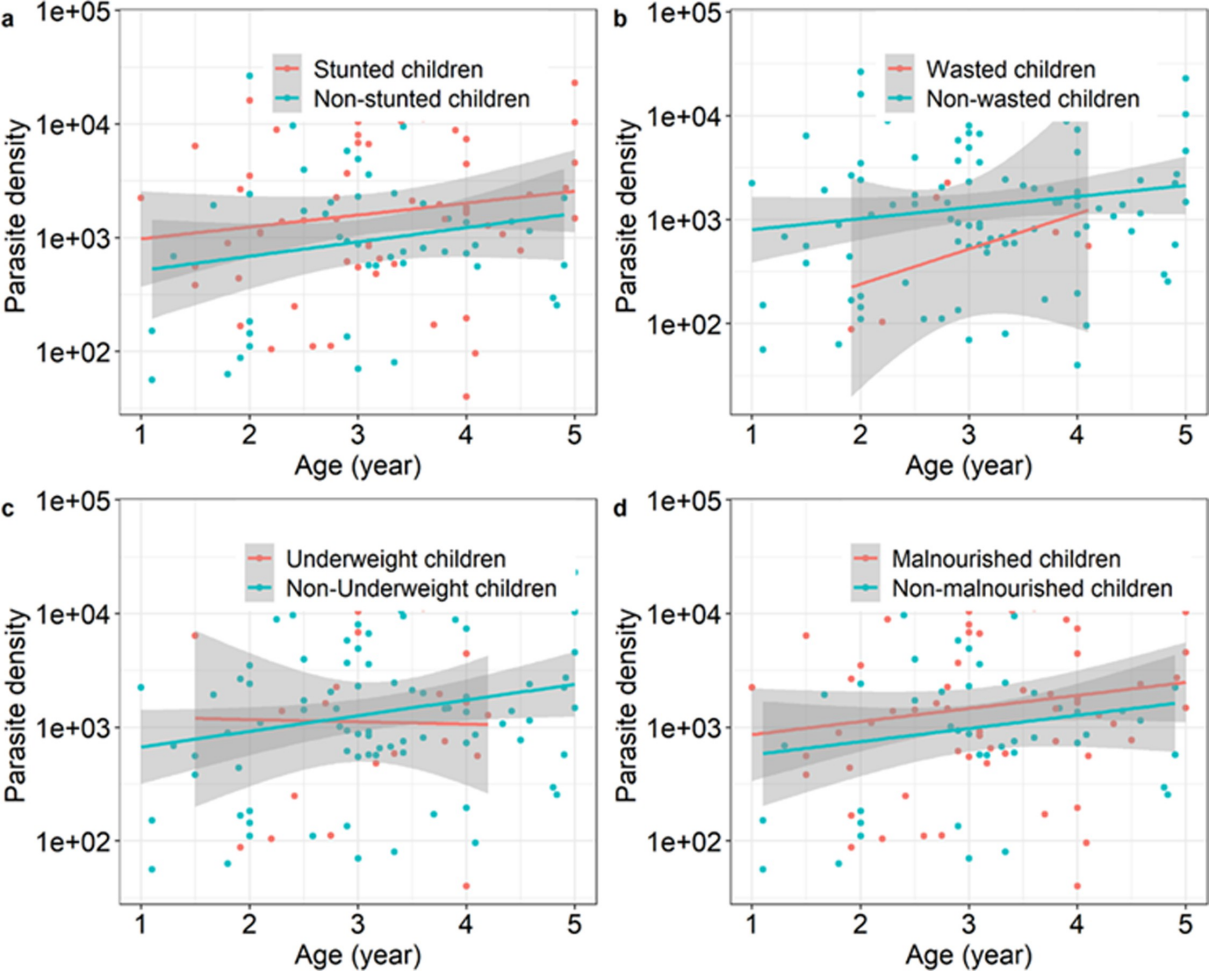
Relationships between parasite density, malnutrition status and age. (a) Parasite density age a function of age in stunted *versus* non-stunted children. (b) Parasite density age a function of age in wasted versus non-wasted children. (c) Parasite density age a function of age in underweighted versus non- underweighted children. (d) Parasite density as a function of age in malnourished (i.e. stunting or wasting or underweight) children versus children with adequate nutritional status.

Although there was no difference in parasite density between underweight and non-underweight children (*X*^*2*^_1_ = 0.48, p = 0.48, Fig 3C) and between undernourished and nourished children (*X*^*2*^_1_ = 1.7, p = 0.19, Fig 3D), stunted children tended to display slightly higher parasite density than non-stunted children (*X*^*2*^_1_ = 3.2, p = 0.07, Fig 3A), and wasted children showed lower parasite density than unwasted children (*X*^*2*^_1_ = 5, p = 0.026, Fig 3B). Age was not a significant predictor for any of the four nutritional parameters studied (Fig 3) and no interaction between age and nutritional parameters on parasite density was found (Fig 3).

**Fig 4:**
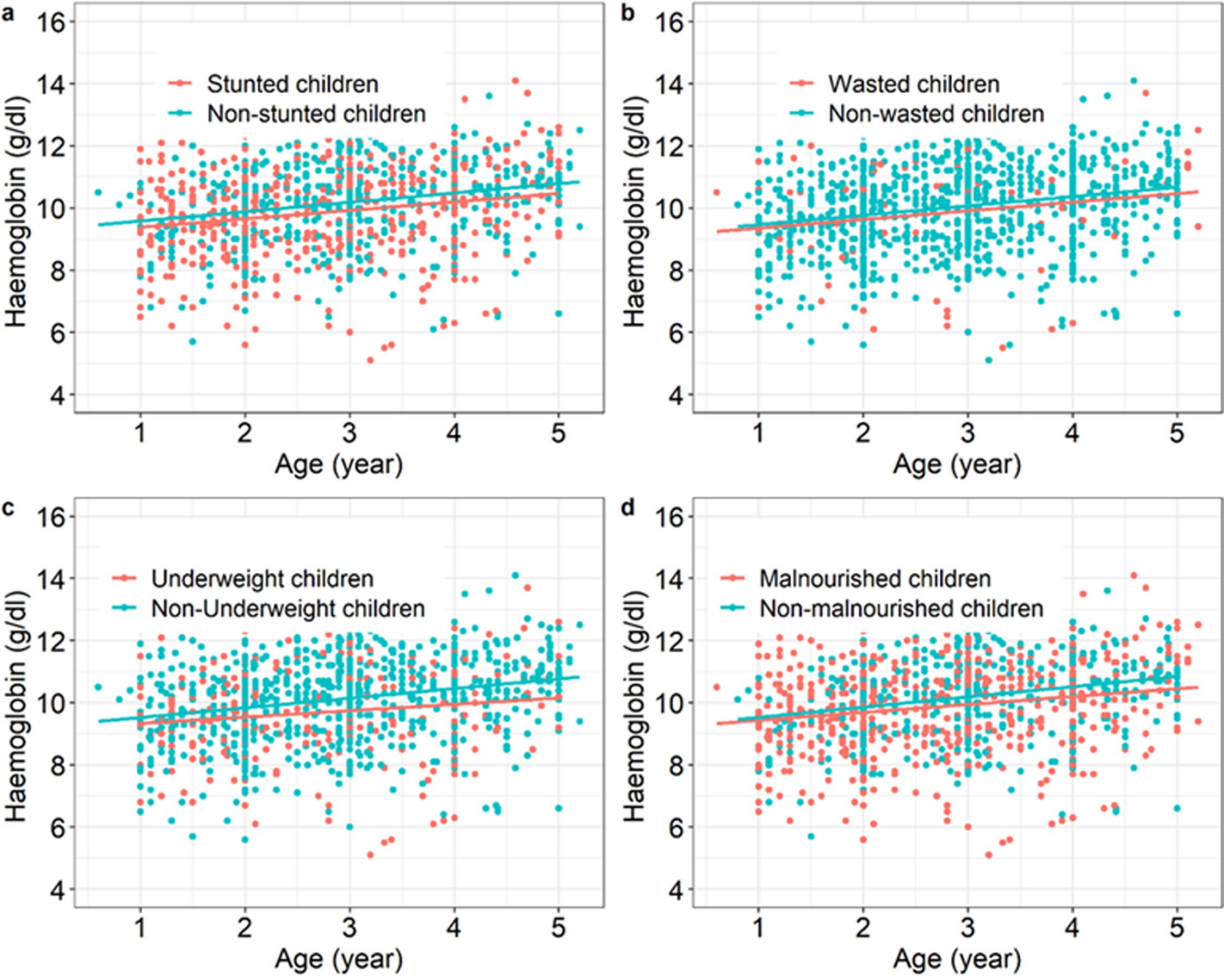
Relationships between Haemoglobin level (g/dl), age and nutritional status. (a) Hb level as a function of age in stunted versus non-stunted children. (b) Hb level as a function of age in wasted *versus* non-wasted children. (c) Hb level as a function of age in underweighted versus non-underweighted children. (d) Hb level as a function of age in undernourished (i.e. stunting or wasting or underweight) children versus children with adequate nutritional status.

Mean haemoglobin level was estimated at 10.0 ±1.4 g/dL. Hb levels of stunted, underweighted and malnourished children were lower than that of their respective counterparts with adequate status (stunting: F_1,1053_ = 8.83, p = 0.003, Fig 4A, underweight: F_1,1053_ = 14, p<0.001, Fig 4C, malnourished: F_1,1053_ = 7.6, p = 0.006, Fig 4D). There was no Hb difference between wasted and unwasted children (F_1,1053_ = 0.9, p = 0.33, Fig 4B). There always was a positive relationship between Hb levels and age and no interaction with nutritional status (Fig 4).

## Discussion

A majority of children under SMC coverage were undernourished at the beginning of the SMC campaign in the health district of Nanoro. This high prevalence of undernutrition is not surprising because this period corresponds to the period of food shortage in rural settings of Burkina Faso. In such context, whether this high malnutrition prevalence could affect the effectiveness of SMC intervention remain poorly assessed. However, the plausibility that malnutrition would reduce SMC drug absorption (AQSP) seems obvious and this could negatively impact on the effectiveness of SMC intervention. More importantly, this situation suggests the necessity of packaging SMC with synergetic intervention against malnutrition. A previous study reported the beneficial effect of the addition of nutrition interventions to reduction of malaria [19].

Expectedly, haemoglobin level represented independent risk factors for malaria infection as anaemia is a symptom of malaria and severe anaemia ranks among the leading severity criteria of malaria infection [26]. Hb level was unexpectedly higher in female than in male children yet gender related difference in Hb level is normally observed in adolescence [27].

As the age of the children increased, an improvement in their nutritional status was observed. A previous study reported an increase in the prevalence of undernutrition as children grow older [28]. These controversial findings could be explained by the fact that the current study included children up the five years old *versus* 24 months old in this previous study and this post-weaning period seems critical for children if the introduction of complementary foods is not adequately performed. This means that malaria infection results in worsening of nutritional status in younger children due probably to weight loss resulting from the decreased food intake during the infection. Indeed, infection by *Plasmodium* was pointed out as a risk factor of malnutrition in young children [29, 30].

Malnourished children had higher parasite densities than children with adequate nutritional status corroborating previous reports [31, 32]. Though the detected infections were mostly asymptomatic, this may increase the susceptibility of malnourished children to subsequent clinical malaria episode [10, 11] as malnutrition is known as a cause of immune deficiency in children. Controversial findings that malnourished children had lower parasitaemia than children with adequate nutritional status was previously reported [33, 34].

However, regarding undernutrition type, this was true for stunted children and underweighted young children, while wasted children appeared to have lower parasite densities than children with ANS. This could result in the protective effect of wasting regarding malaria infection [15]. Similarly, increased susceptibility of stunted children to malaria infection was previously reported in the Gambia [11, 32]. In rural Africa, stunting in children result frequently from micronutrient deficiencies. In such context, this finding also suggest that micronutrients supplementation would be an adequate strategy for tackling malaria and malnutrition in young children in Africa. However, previous study reported conflicting results that stunting or wasting was not associated with increased risk of malaria infection [35].

RPR and MPR were relatively higher at the start of SMC campaign suggesting the need to start the campaign earlier. Therefore, the curative effect of SMC based on AQ need to be as efficacious to clear existing parasitaemia. However, there is currently no recommended way for the assessment of the efficacy AQSP. Interestingly in this study, the more AQSP tablets ingested, the less likely it was to be positive by RDT or microscopy suggesting a good efficacy of AQSP.

Interestingly, the majority of children slept under bed nets the night preceding the day of the survey. Even most of the non-compliant population had yet the LLIN available in their house, justifying the importance of mass freely distribution campaign of LLIN every two years by the ministry of health. Therefore, in addition to sustaining this intervention, continued community sensitization is necessary to reduce the number of non-compliant population.

## Conclusion

In the Nanoro health district, children who received Seasonal Malaria Chemoprevention (SMC) are mostly undernourished during the period of SMC delivery, suggesting the need for combining the SMC with synergistic interventions against malnutrition to achieve best impact.

## Supporting information

S1 Data Data set for baseline malarial and nutritional profile of children under SMC coverage in the health district of Nanoro, Burkina Faso. (XLSX)
